# Hemoglobin enhances miRNA-144 expression and autophagic activation mediated inflammation of microglia via mTOR pathway

**DOI:** 10.1038/s41598-017-12067-2

**Published:** 2017-09-19

**Authors:** Zhenyu Wang, Bangqing Yuan, Fenlan Fu, Shaokuan Huang, Zhao Yang

**Affiliations:** 1 Department of rehabilitation medicine, Yongchuan Hospital, Chongqing Medical University, Chongqing, 402160 China; 2 Department of Neurology, Yongchuan Hospital, Chongqing Medical University, Chongqing, 402160 China

## Abstract

Intracerebral hemorrhage promotes autophagic activation of microglia and enhances neuroinflammation. MiRNAs are key factors to autophagy, contributed to negatively and posttranscriptionally regulate gene expression and function. However, the specific miRNAs involved in the intracerebral hemorrhage mediated microglia autophagic activation are unidentified. In this experiment, microglia was treated with hemoglobin. And then, miRNA-144 expression, autophagic activation and inflammation of microglia were detected. In addition, the mTOR target of miRNA-144 and its regulation were identified. Our data demonstrated that hemoglobin promoted miRNA-144 expression and autophagic activation mediated inflammation. Additionally, miRNA-144 targeted mTOR by directly interacting with the 3′ untranslated regions (UTRs), mutations of the binding sites abolish the miRNA-144 responsiveness. Overexpression of mTOR decreased autophagic activation and inflammation of microglia. Therefore, our results suggested that miRNA-144 contributed to hemoglobin mediated autophagic activation and inflammation of microglia via mTOR pathway. And miRNA based treatment provided novel therapeutical strategy for intracerebral hemorrhage.

## Introduction

Intracerebral hemorrhage (ICH) is a vital subtype of stroke, leading high morbidity and mortality^1–3^. The incidences of ICH bring damage to the brain function, results in cognitive decline or brain injury^4^. The previous experiments have indicated that microglia mediated inflammation contributed to the pathology progress of ICH^5–7^.

Autophagy is a primary cellular process which exists in majority of cell types of the mammal organism, and regulated by different stress conditions leading to inflammatory response^8–10^. Autophagy plays an important role in innate and adaptive immune cells, therefore, it activation affect antibacterial response, immune defense and inflammatory responses^11–13^.

miRNAs are noncoding RNAs (ncRNAs), including about 20–25 nucleotides. These small ncRNAs control target gene expression via posttranscriptional function^14–16^. In addition, miRNAs also play an important role in pretranslational and cotranslational progress to perform direct or indirect role on translational mechanism^17–19^. In normal condition, miRNAs contribute to cell differentiation, proliferation, metabolism and death. In pathological conditions, abnormal miRNA expression leads disease via regulating different genes^20–22^.

The related studies demonstrated that miR-144 inhibited proliferation and promoted autophagy of A549 and H460 cells by targeting TIGAR^23^. In addition, VEGF-activated miR-144 regulated autophagic activation of prostate cancer cells against Cisplatin^24^. However, the role of miRNA-144 in the autophagy and inflammatory response of microglia in ICH has not been well identified. In this experiment, we found that hemoglobin promoted miRNA-144 levels, leading to autophagy and inflammatory response of microglia.

## Materials and Methods

### Antibodies and Reagents

The GFP-MAP1LC3B plasmid was kindly offered by Dr. Tamotsu Yoshimori (Department of Cell Biology, National Institute for Basic Biology, Presto, Japan). 3-Methyladenine (3-MA, a synthetic intermediate and a cell-permeable autophagic sequestration blocker that protects cerebellar granule cells from apoptosis post serum/potassium deprivation. The final concentration is 5 mM), Bafilomycin A1 (Baf A1, an inhibitor of maturation of autophagic vacuoles by inhibiting fusion between autophagosomes and lysosomes. The final concentration is 10 nM), or activators (rapamycin, Rapa, an inducer of the autophagy pathway by inhibiting the mammalian target of rapamycin mTOR pathway. The final concentration is 100 nM) were purchased from Sigma; antibodies against MAP1LC3B (L7543) were obtained from Sigma.

### Cells

Primary hippocampal microglial cells were isolated from glial cultures prepared from newborn (less than 24 hours old) Sprague-Dawley (SD) rats(Laboratory Animal Center, Chongqing Medical University, Chongqing, China). Glial cells were cultured in 75 cm^2^ flasks for 14 days in DMEM/F12 (Gibco BRL, Grand Island, NY, USA) supplemented with 10% FCS(Hyclone, Logan, UT, USA), 100 U/ml penicillin and 100 mg/ml streptomycin. Microglia were isolated from primary mixed glial cell cultures on day 10 by shaking the flasks overnight at 300 rpm on a rotary shaker at 37 °C. Microglia were cultured in DMEM/F12 (Gibco BRL, Grand Island, NY, USA) supplemented with 10% FCS(Hyclone, Logan, UT, USA), 100 U/ml penicillin and 100 mg/ml streptomycin. The purity of the microglial cultures was assessed as over 90%, using a CD11b antibody. Cells were cultured for 2 days before treatment. The Chongqing Medical Experimental Animal Care Committee approved the protocol for this study, and all animal experiments were conducted in accordance with the National Institutes of Health Guidelines for the Care and Use of Laboratory Animals.

### Cell treatment

Microglia (1 × 10^5^) was cocultured with 10 µl PBS or hemoglobin (20 μM) for 24 h. After then, the supernatants were removed and further analyzed for cytokine production with ELISA.

### qRT-PCR

For mRNA quantification, total RNA was isolated from cells 24 hours after transfection and transcribed with SuperScript II and anchored oligo-d(T)20 primer (Invitrogen). Amplification and quantification of cDNA was carried out with SYBR Green ROX Mix (Abgene, Epsome) according to the manufacturer’s protocol. qRT-PCR was carried out in 7900 HT Fast Real-Time PCR System (Applied Biosystems). For each of the selected miRNAs, real-time PCR measurements were performed to obtain a mean CT value for each sample. The CT values of the different samples were compared using the 2- ^ΔΔ^ CT method. The gene expression levels were normalized to β-actin levels. The sequences of primers used were shown as following: IL-6, 5′-AGCATACA GTTT GT GG ACATT-3′(forward), 5′-CAACATTCATATTGCCAGTTCT -3′(reverse); IL-1β, 5′-CAGGCAACCACTTACCTATTTA-3′(forward),5′-CCATA CACAC GGACAACAACTAGAT-3′(reverse); TNF-α, 5′-CGAGTGACAAGCCT GTAGC -3′(forward); 5′-TACTTGG GCAGAT TGACCTCA -3′(reverse); 5′-TCGTGCCTGTCTGATTCTC -3′(forward); mTOR, 5′-GATTCATGCC CTTCTCTTTGG-3′(reverse); miR144, 5′-GCGGGCGGATATCATCATAT -3′(forward); 5′-GCTACTT AGCG CGCTACTT ACTGGACA CTGG CA GTCGCGAACTGTAAG-3′(reverse);GADPH, 5′-CAT GG TCTA CATG TTCC A GT-3′(forward); 5′-GGC TAAG CAGTTGGTGGT GC -3′ (reverse).

### Western blotting

Cells were harvested 24 hours after transfection and lysed in 50 mmol/L Tris-HCl, pH 7.5; 150 mmol/L NaCl; 1% NP-40, containing Complete Mini protease inhibitor mixture (Roche Diagnostics). Protein extracts were electrophoresed on 4% to 12% linear gradient Bis-Tris ready gels (Invitrogen) and transferred to Immobilon-P polyvinylidene fluoride membranes (Millipore). Membranes were probed with antibodies specific for LC3-B (1:1,000, 2775S; Cell Signaling, Inc.) and actin (1:2,500, CB1001; Calbiochem), and subsequently with horseradish peroxidase-coupled anti-rabbit or anti-mouse antibodies (both 1:2,500; Cell Signaling Inc.). Specific bands were visualized with ECL Blotting Detection Reagents (Amersham Biosciences) and quantified with Image J software.

### Construction of luciferase plasmids and reporter assay

The 3′-untranslated region (UTR) of the mouse mTOR was synthesized from Invitrogen. The PCR product was cloned downstream of the renilla luciferase stop codon in pMIR report vector (Ambion, USA), named 3′UTR- mTOR. We constructed single-mutant luciferase reporter vectors (3′UTR mut-mTOR) by site-directed mutagenesis. In 6-well plates, 2.0 × 10^5^ microglia per well were plated. When the cells were 50% confluent, they were transfected with 80 ng of the luciferase reporter plasmid, 40 ng of the pRL-TKRenilla-luciferase plasmid (Promega Corp., Madison, WI, USA), and the indicated RNAs (final concentration 20 nmol/l). A renilla luciferase vector (pRL-TK) was used to normalize differences in transfection efficiency. Cells were harvested 24 hours after transfection and assayed using Luciferase Assay System (Promega, Madison, WI) according to the manufacturer’s handbook. Each transfection was repeated twice in triplicate. In addition, the mTOR overexpressing plasmids with the CMV promoters were purchased form the Santa Cruz company.

### Oligonucleotide transfection

Microglia were transduced with 50 nmol/l of miRNA mimics and with 50 nmol/l of RNA duplexes according to the manufacturer’s protocol (Applied Biosystems, Carlsbad, CA). After cocluture for 6 hr, the fresh medium was added. And then, 24 h after transfection, microglia were collected and utilized for further analysis such as RT-PCR or western blot. The microglia were trypsinized to identified the viability over 90%.

### Transmission Electron Microscopy

Microglia were fixed using 2.5% glutaraldehyde, postfixed in 1% osmium tetroxide and 1% potassium, and embedded in Quetol 812 (Nisshin EM). Thick sections were stained with toluidine blue for light microscopy. Ultrathin sections were stained with uranyl acetate and lead nitrate and observed using a Hitachi H-7500 electron microscope. An autophagosome is identified as a spherical structure with double layer membranes. The outer membrane of an autophagosome fuses with a lysosome to form an autolysosome. The lysosome’s hydrolases degrade the autophagosome delivered contents and its inner membrane.

### Fluorescence confocal microscopy

GFP-MAP1LC3B transduced microglia grown on chamber slides were treated with hemoglobin or PBS for 6 h. The cells were fixed in 4% paraformaldehyde for 10 min at room temperature and washed with PBS. The slides were analyzed under a laser scanning confocal fluorescence microscope (Olympus FV300). Images were analyzed for the presence of cytoplasmic GFP-LC3B+ puncta by means of the BD Attovision software (BD Imaging Systems).

### Acridine orange staining

Autophagy is characterized by the formation and promotion of acidic vesicular organelles (AVOs). The intensity of the red fluorescence is proportional to the degree of acidity. Following treatment, Microglia were grown on the cover slips in 6-well plate. Then, the microglia were incubated with 1 mg/mL acridine orange (Sigma) for 15 min. Pictures were obtained with a fluorescence microscope. Then, cells were trypsinised and analysed by FACScan cytometer with MetaMorph software.

### Monodansylcadaverine (MDC) staining

MDC, a specific marker for autophagic vacuoles, was used to examine whether hemoglobin induced microglia autophagy. Following treatment, Microglia were grown on the cover slips in 6-well plate. The cells were washed with ice-cold PBS, and incubated with 50 μM of MDC at 37  °C for 30 min. The stained cells were washed, fixed with 4% paraformaldehyde, and analyzed by FACScan cytometer with MetaMorph software.

### ELISA assay

The IL-6, IL-1β and TNF-α expression levels in the in the culture supernatants were detected by sandwich ELISA method using a commercial ELISA kit from R&D Systems (Minneapolis, MN, USA) according to the manufacturer’s instructions. Microglia (0.5 × 10^6^) plated in 24-well plates were cultured with hemoglobin for 6 h. The culture supernatants were collected and assayed by ELISA. Based on the color reaction of the cytoplasm extract and antibodies, the absorbance values were determined at 450 nm on a Microplate Reader.

### Statistical analysates

The differences between groups were determined with the two-way analysis of variance (ANOVA) using SPSS 13.0 software. Differences were declared significant at *P < 0.05.

## Results

### Hemoglobin promoted autophagy of microglia

**Figure 1:**
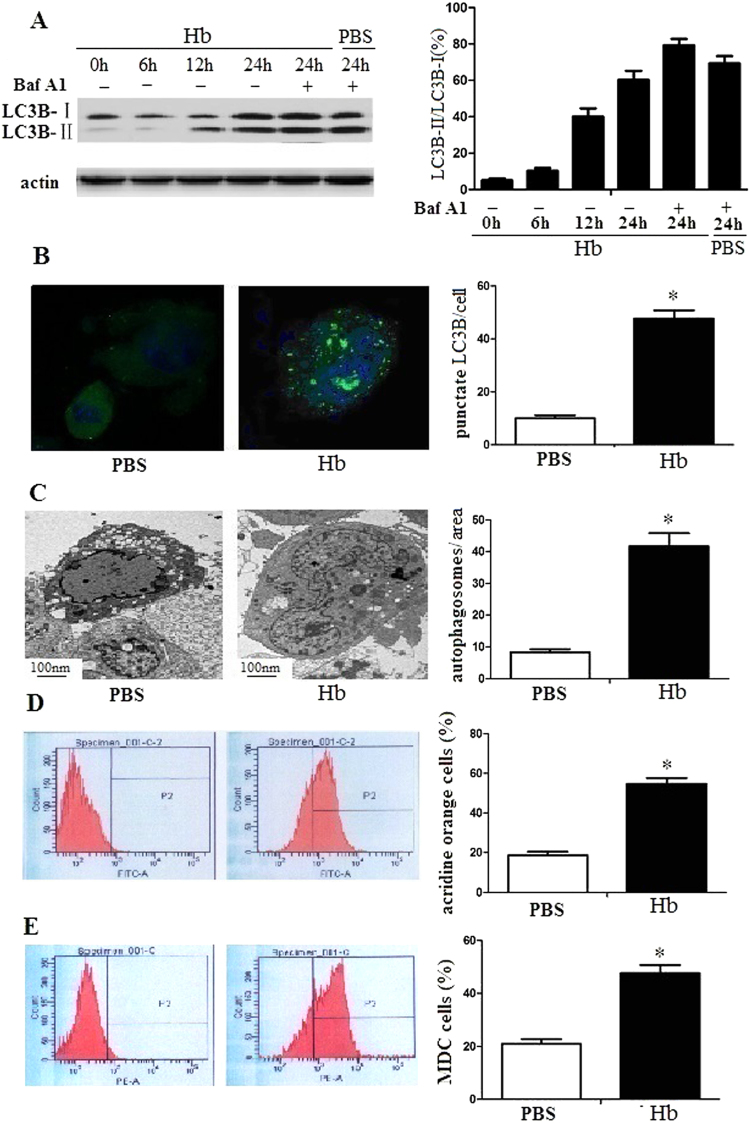
Hemoglobin stimulated microglia autophagy. (**A**) Autophagic flux of microglia was generated by hemoglobin. The cells were cocultured with hemoglobin for 0–24 h with or without. Baf A1 (10 nM). Control groups were cocultured with PBS. (**B**) Microglia were transduced with a GFP-MAP1LC3B plasmid. After 24 h, microglia were elicited by hemoglobin for 24 h. After then, microglia were detected by confocal microscopy. The number of GFP-MAP1LC3B puncta of each microglia was calculated by computer. (**C**) Ultrastructural changes of microglia elicited by hemoglobin or PBS were detected by TEM. (**D**) Microglia were treated with hemoglobin or PBS for 24 h and stained with 1 mg/ml acridine orange or 50 mM MDC for 15 min. After stimulation, the number of positive microglia were detected by flow cytometry. The bar chart shows an increase in mean fluorescent intensity. Data are presented as the mean ±SD of three independent experiments. P < 0.05.

To analyze whether hemoglobin could promote autophagy of microglia, we detected the MAP1LC3B marker (an accurate indicator of autophagy) expresson of microglia. We found that the ratio of LC3B-II to LC3B-I expression increased after hemoglobin treatment compared with control cells (Fig. 1A). In addition, Baf A1 increased further MAP1LC3B-II accumulation of microglia after 24 hrs, demonstrating that hemoglobin promoted microglia autophagic flux. To further detect that hemoglobin induced autophagy of microglia, we utilized a GFP-MAPLC3B puncta formation assay to analyze autophagy. The results demonstrated that hemoglobin promoted GFP-MAPLC3B positive puncta of microglia compared with control cells after 24 h (Fig. 1B). TEM analysis also revealed increased number of autophagosomes in the microglia after hemoglobin treatment (Fig. 1C). Acridine orange staining and MDC staining assays had significant changes (Fig. 1D). The results suggested that hemoglobin promoted autophagy of microglia.

### Autophagy contributed to hemoglobin induced microglia inflammation

**Figure 2:**
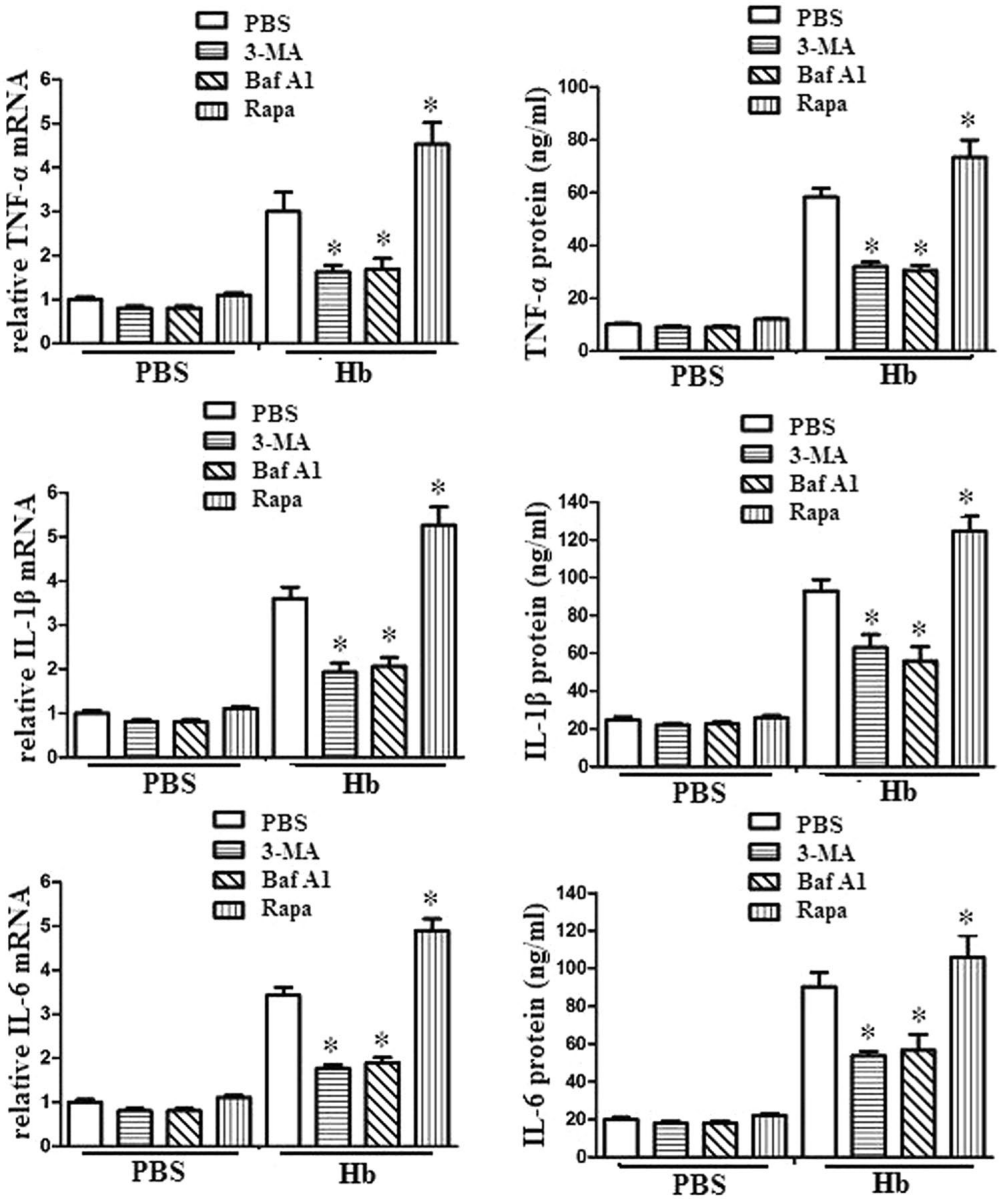
Autophagy was involved in hemoglobin induced microglia inflammation. Microglia (1 × 105) was treated with 100 nM rapamycin, 5 mM 3-MA, 10 nM Baf A1, or the indicated combinations for 24 h. After the treatment, the cells were cultured with 10 µl PBS or hemoglobin for 24 h. we analyzed the inflammatory cytokine expression. The mRNA and protein levels of TNF-α, IL-1β and IL-6 were determined. Experiments performed in triplicate showed consistent results. Data are presented as the mean ±SD of three independent experiments. *P < 0.05.

To exam whether autophagy promoted microglia inflammation after hemoglobin treatment, we cocultured microglia with autophagy inhibitors (3-MA or Baf A1,) or activators (rapamycin, Rapa), and analyzed the subsequent microglia inflammatory response after hemoglobin treatment. The results demonstrated that autophagy inhibitors (3-MA or Baf A1) attenuated microglia inflammatory response after hemoglobin treatment. However, autophagy activators (rapa) promoted inflammatory response after hemoglobin treatment (Fig. 2).

### Hemoglobin induced microglia autophagy by mTOR

**Figure 3:**
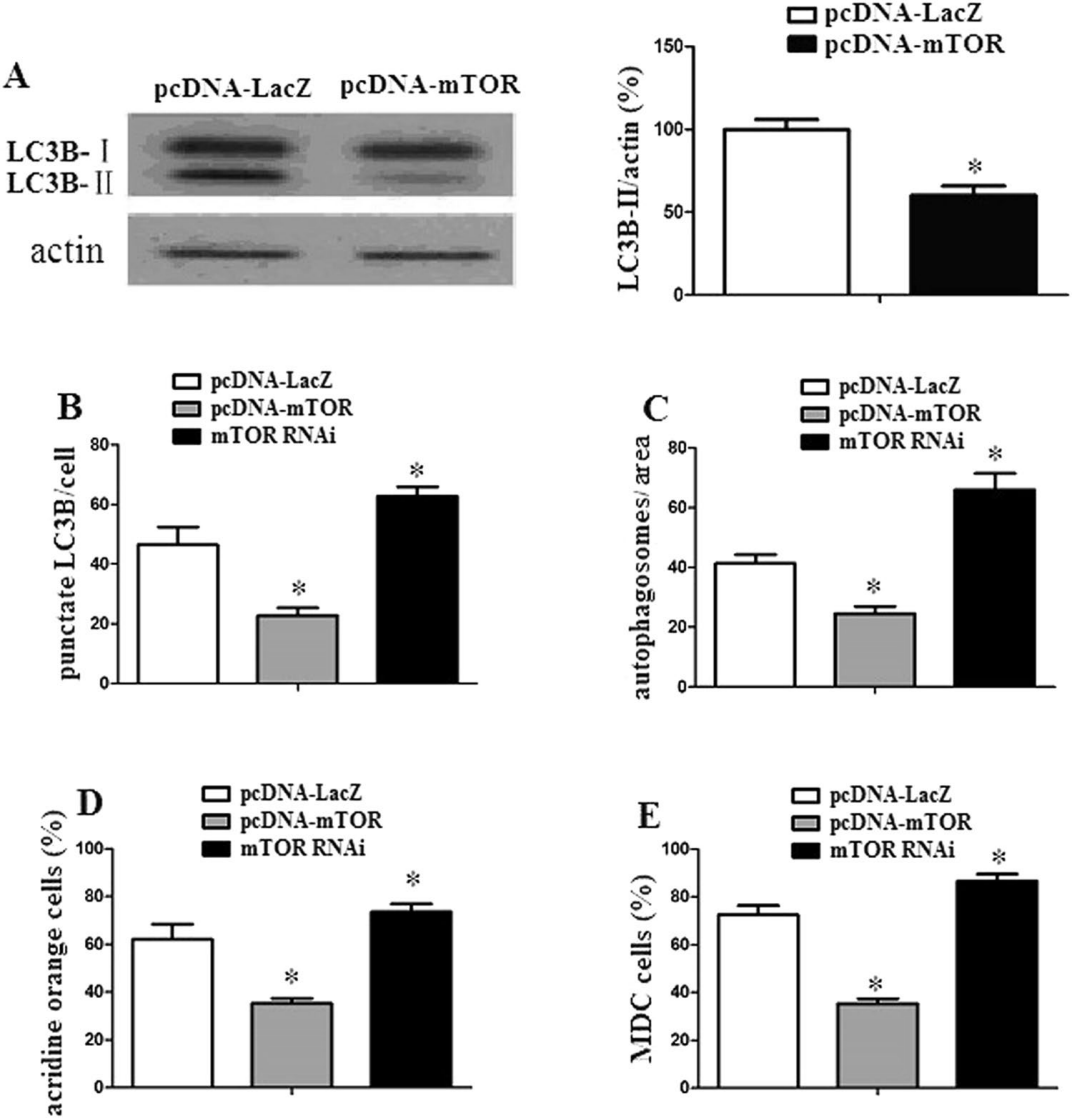
Hemoglobin elicited microglia autophagy via mTOR. (**A**) mTOR attenuated autophagic flux of microglia elicited by hemoglobin. Microglia were transduced with pcDNA-LacZ or pcDNA-mTOR and then stimulated by hemoglobin for 24 h. The ratio of LC3B-II to LC3B-I of microglia was detected by western blot. (**B**) Microglia encoding GFP-MAP1LC3B were transduced with pcDNA-LacZ, pcDNA-mTOR or mTOR RNAi, and then stimulated by hemoglobin or PBS. Microglia were detected by confocal microscopy. The number of GFP-MAP1LC3B puncta of each microglia was calculated. (**C**) Ultrastructural changes of microglia elicited by hemoglobin or PBS were detected by TEM. (**D**) Microglia were treated with hemoglobin or PBS for 24 h and stained with 1 mg/ml acridine orange or 50 mM MDC for 15 min. After stimulation, the number of positive microglia were detected by flow cytometry. The bar chart demonstrates an increase in mean fluorescent intensity. Data are presented as the mean ±SD of three independent experiments. P < 0.05.

Although accumulating studies have reported that mTOR could mediate autophagy in different diseases, it is not clear whether mTOR mediates microglia autophagy induced by hemoglobin. As shown in Fig. 3A, there was a significant decrease in the ratio of LC3B-II to LC3B-I in pcDNA-mTOR transduced microglia following hemoglobin treatment. The formation of GFP-LC3 puncta is another marker of autophagosomes. As shown in Fig. 3B, pcDNA-mTOR decreased the number of GFP-LC3 puncta in microglia after hemoglobin treatment (P < 0.05). TEM analysates also revealed a decrease in the number of autophagosomes in the microglia transduced with pcDNA-mTOR (Fig. 3C). In addition, acridine orange staining and MDC staining assays had significant changes (Fig. 3D). These indicated that mTOR promoted microglia autophagy after hemoglobin treatment.

### Hemoglobin upregulated miRNA-144 expression but downregulated mTOR expression in microglia

**Figure 4:**
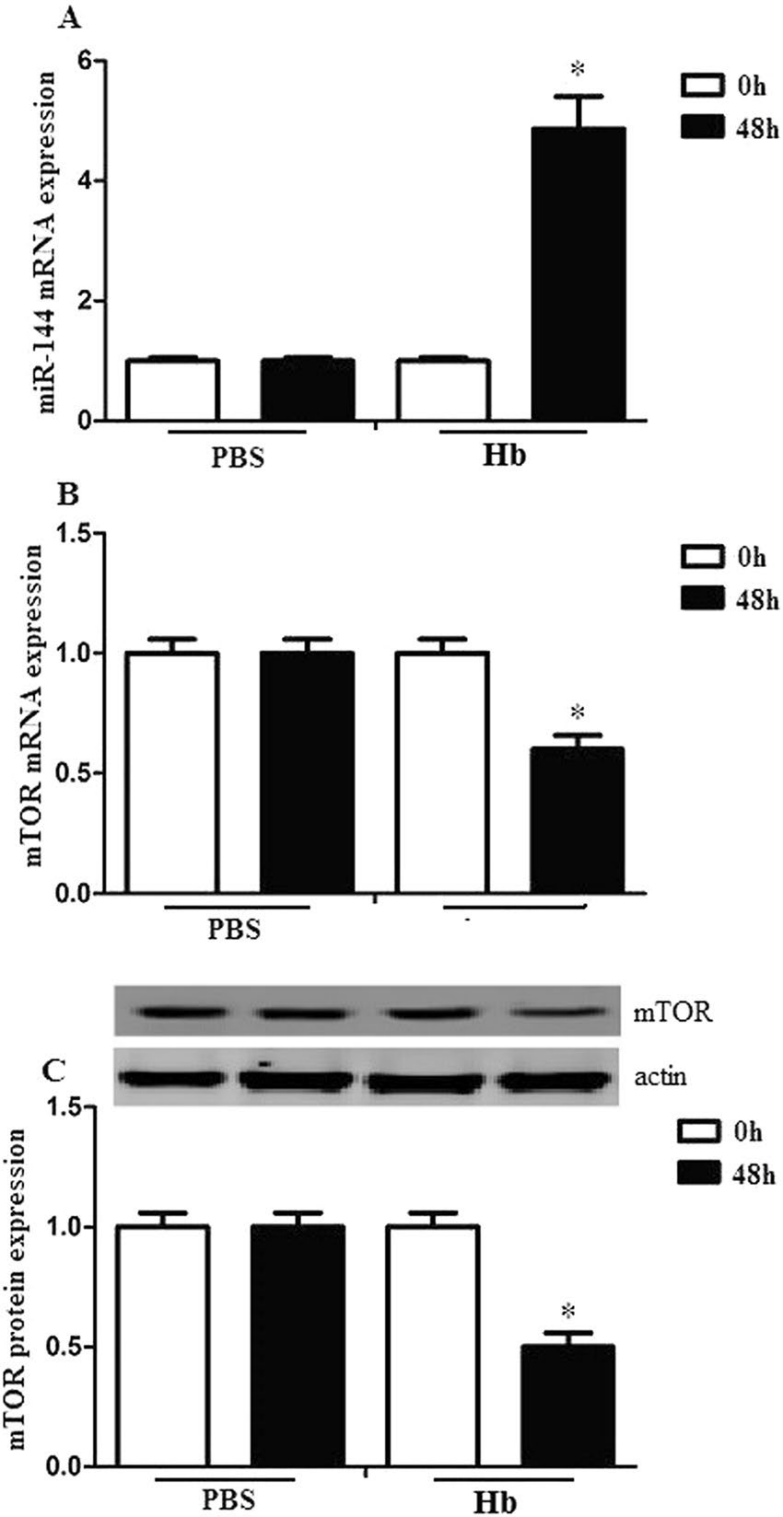
Hemoglobin upregulated miR-144 expression but downregulated mTOR expression in microglia. Microglia (1 × 105) was stimulated with 10 µl PBS or hemoglobin for 48 h. (**A**) miR-144 expression levels were evaluated by quantitative RT-PCR. (**B**–**C**) mTOR mRNA and protein expression levels were evaluated by RT-PCR and western blots. Experiments performed in triplicate showed consistent results. Data are presented as the mean ±SD of three independent experiments. *P < 0.05.

We measured miRNA-144 and mTOR expression of microglia 48 hs after hemoglobin or PBS treatment. We found that miRNA-144 expression significantly increased after hemoglobin treatment. On the contrary, mTOR mRNA and protein expression decreased after hemoglobin treatment (Fig. 4). These results demonstrated that hemoglobin upregulated miRNA-144 expression but downregulated mTOR expression in microglia.

### miR-144 regulated mTOR expression in microglia

**Figure 5:**
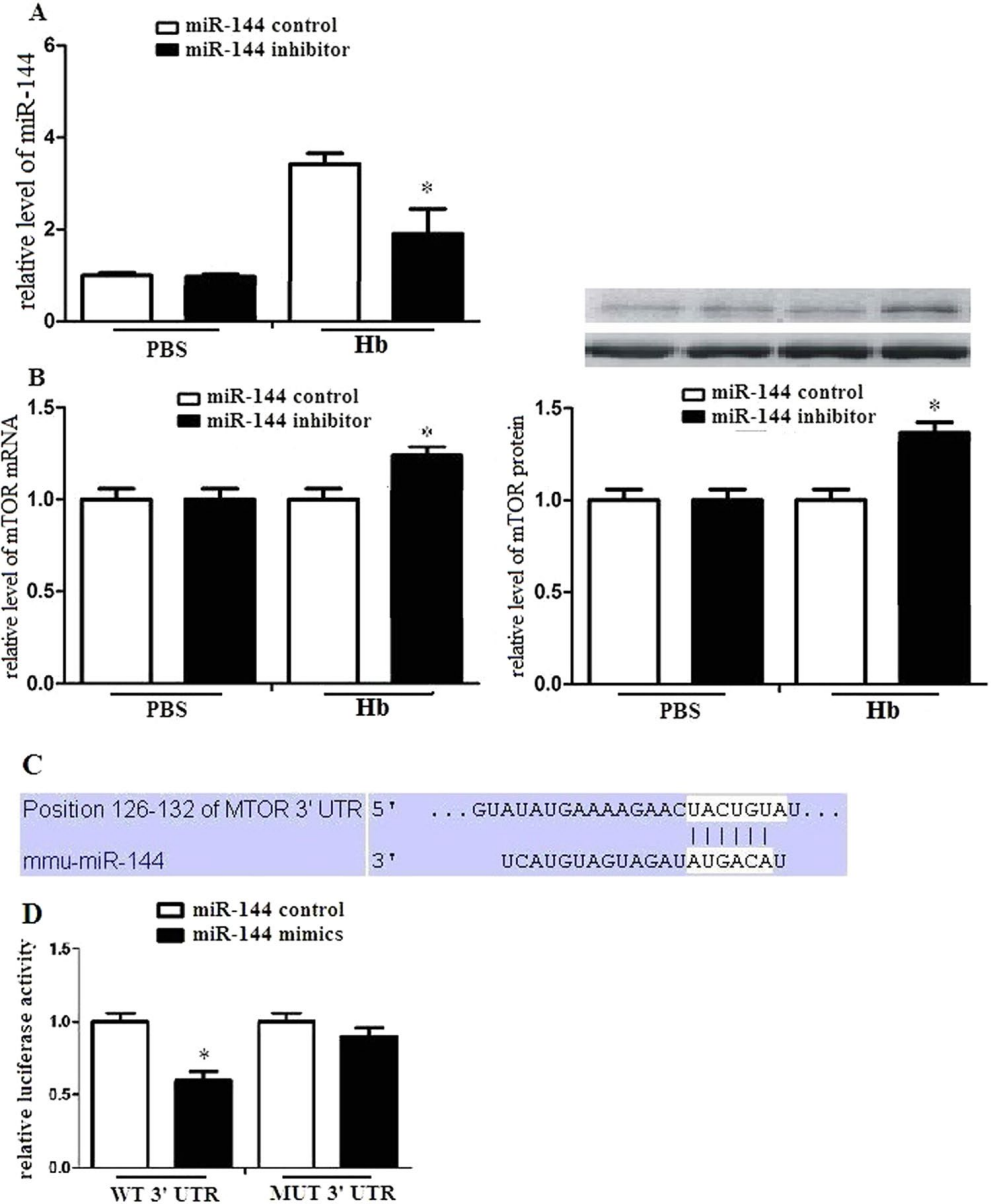
miR-144 regulated mTOR expression in microglia. (**A**) Microglia was transduced with miR-144 inhibitor or miR-144 control. After 24 h, cells were harvested, and miR-144 expression was evaluated by RT-PCR. (**B**) Microglia was transduced with miR-144 inhibitor or miR-144 control, and then was stimilated with PBS or hemoglobin After 24 h, cells were harvested, and mTOR mRNA and protein expression were evaluated by RT-PCR and western blots. (**C**) A mTOR 3′UTR fragment containing wild-type or mutant miR-144- binding sites was cloned downstream of the luciferase reporter gene. The region of the mTOR mRNA 3′ UTR predicted to be targeted by miR-144 as indicated. (**D**) Luciferase activity assays using reporters with wild- type or mutant mTOR 3′ UTRs were performed after cotransfection with miR-144 mimics or control in microglia. The luciferase activity of the control transfection in each experiment was used to normalize the data, and the luciferase activity of the control transduction was set equal to 1. Experiments performed in triplicate showed consistent results. Data are presented as the mean ±SD of three independent experiments. *P < 0.05.

To detect the relationship of miR-144 and mTOR, we transduced microglia with miR-144 inhibitor or miR-144 control. We found that transfection of miR-144 inhibitor attenuated miR-144 mRNA expression (Fig. 5A). We further transduced microglia with miR-144 inhibitor or miR-144 control, and then treated the microglia with hemoglobin. Microglia transduced with miR-144 inhibitor had dramatically improved mTOR expression (Fig. 5B), which suggests that miR-144 inhibits hemoglobin induced mTOR expression in microglia.

### mTOR was a direct target of miR-144 in microglia

We made a hypothesis that mTOR was a direct target of miR-144 in microglia. The target prediction program TargetScan (www.targetscan.org) predicted the 3′-UTR of mTOR mRNA contained a putative miR-144 target sequence (Fig. 5C). TargetScan predicted biological targets of miRNAs according to the presence of conserved 8mer, 7mer, and 6mer sites that matched the seed region of each miRNA. In mammals, predictions are ranked based on the predicted efficacy of targeting as calculated by cumulative weighted context scores of the sites. As an option, predictions are also ranked by their probability of conserved targeting (PCT, Friedman *et al*., 2009). We detected the relationship of mTOR and miR-144 by a dual-luciferase reporter system. Co-transduction with miR-144 mimics significantly suppressed the activity of a firefly luciferase reporter coding wild-type mTOR 3′-UTR. However, miR-144 mimics could not suppress the activity of a firefly luciferase reporter with a mutated mTOR 3′-UTR (Fig. 5D). These results indicated that miR-144 inhibited mTOR expression via directly binding target sites in the mTOR 3′-UTR.

### miR-144 promoted inflammatory response of microglia via mTOR

**Figure 6:**
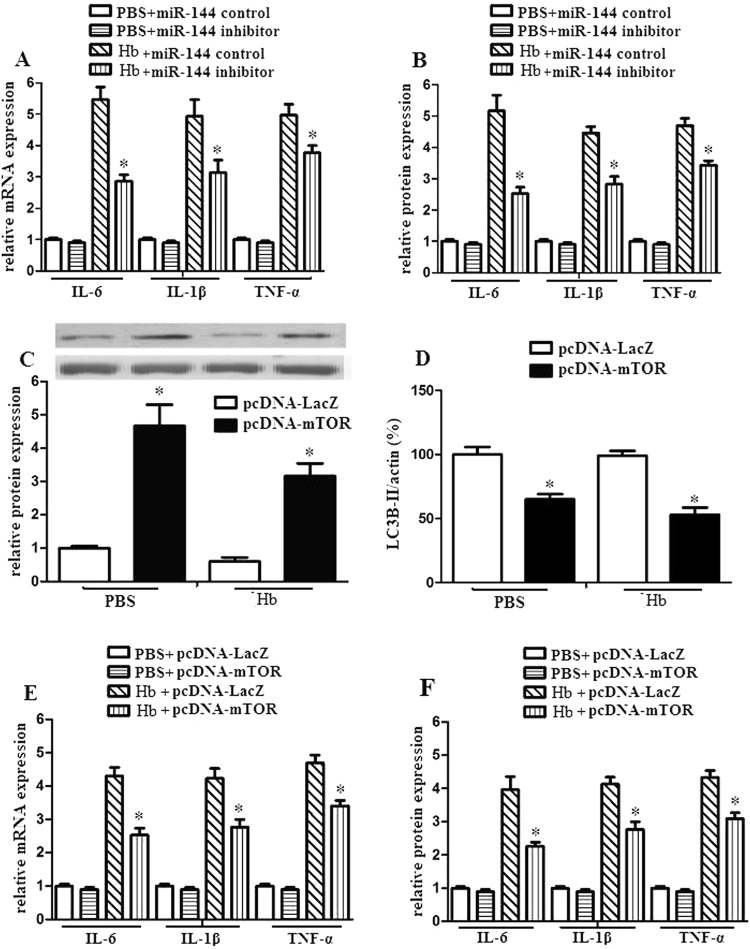
miR-144 promoted the downstream production of proinflammatory mediators via mTOR in microglia. (**A**–**B**) Microglia were transduced with miR-144 inhibitor or miR-144 control. And then, microglia (1 × 105) were stimulated with PBS or hemoglobin for 24 h. IL-6, IL-1β and TNF-α mRNA and protein expression were evaluated by RT-PCR and ELISA. (**C** and **D**) Microglia were transduced with pcDNA-mTOR or pcDNA-LacZ and then exposed to PBS or hemoglobin. After 24 h, mTOR protein expression and autophagic activation were analyzed using western blot. (**E** and **F**) Microglia were transduced with pcDNA-mTOR or pcDNA-LacZ and then exposed to PBS or hemoglobin. After 24 h, IL-6, IL-1β and TNF-α mRNA and protein expression were evaluated by RT-PCR and ELISA. Experiments performed in triplicate showed consistent results. Data are presented as the mean ±SD of three independent experiments. *P < 0.05.

We detected the effects of miR-144 on hemoglobin treated microglia. The results demonstrated that miR-144 inhibitor attenuated inflammatory mediator expression of hemoglobin treated microglia (Fig. 6A–B).

To identify whether miR-144 decreases autophagic activation and promotes inflammatory response of microglia via mTOR, we utilized pcDNA-mTOR to promote mTOR expression and observed the autophagic activation and inflammatory response of microglia. We found that pcDNA-mTOR significantly increased mTOR expression and decreased autophagic activation (Fig. 6C and D). In addition, upregulation of mTOR significantly decreased the downstream production of proinflammatory mediators (Fig. 6E and F). These data demonstrated that miR-144 promotes inflammatory response of microglia via mTOR.

## Discussion

ICH is characterized as nontraumatic bleeding, which happens in the brain parenchyma. It is a critical emergency with poor consequence and high morbidity^25–27^. ICH occurs within minutes to hours from the beginning of bleeding, leading to primary mechanical injury and secondary injury, which initiates cytotoxic, oxidative, and inflammatory roles^28–30^. ICH regulates divergent inflammatory factors, such as TNF-α and IL-1β, which initiating microglial activation and other immune cell infiltration^31–33^. Hemoglobin is a key factor to inflammation that leaks from damaged erythrocytes in the ICH^34^.

Autophagy is a fundamental cellular process which is involved in lysosomes mediated destroy and digestion of intracellular ingredients^35–37^. This course plays a crucial role in metabolic homeostasis for keeping cellular balance and avoids the accumulation of misfolded proteins and damaged intracellular ingredients^38–40^. Autophagy also contributes to numerous acute and chronic neurological diseases, such as brain trauma, vascular dementia, stroke and other neurodegenerative diseases^41–43^.

miRNAs are small noncoding RNAs which control the gene expression at the post-transcriptional level by attenuating translation or degrading mRNA^44–46^. A single miRNA controls various target genes, and plays a crucial role in the innate and adaptive immune system. Therefore, abnormal miRNA expression will lead to serious immune related disease^47^. For example, lack of miR-124 leads neuron injury and inflammatory response in stroke. On the contrary, administration of miR-124 represents a novel therapeutical effect on stroke^48^. In the previous studies, we identified that ICH downregulated miR-367 expression but upregulated IRAK4 expression in primary microglia. We also demonstrated that miR-367 suppressed IRAK4 expression and the downstream production of proinflammatory mediators. However, the specific miRNA involved in the autophagy of microglia has been few reported.

Firstly, to analyze whether hemoglobin could promote autophagy of microglia, we detected the MAP1LC3B marker expresson of microglia. We found that hemoglobin promoted autophagy of microglia. To exam whether autophagy promoted microglia inflammation after hemoglobin treatment, we analyzed the subsequent microglia inflammatory response after hemoglobin treatment. The results demonstrated that autophagy inhibitors attenuated microglia inflammatory response after hemoglobin treatment. However, autophagy activators promoted inflammatory response after hemoglobin treatment. To detect whether mTOR mediates microglia autophagy induced by hemoglobin, we detected the relationship between mTOR and microglia autophagy. The results demonstrated that mTOR promoted microglia autophagy after hemoglobin treatment. Lastly, to detect the relationship between miR-144 and mTOR, we identified that miR-144 inhibited mTOR expression via directly binding target sites in the mTOR 3′-UTR. And miR-144 promoted inflammatory response of microglia via mTOR.

In summary, our data suggested that miRNA-144 contributed to hemoglobin mediated autophagic activation and inflammation of microglia via mTOR pathway. And miRNA based treatment provided novel therapeutical strategy for ICH.
